# Supplementary material for: Patterns of Intron Gain and Loss in Fungi
Source: PLoS Biol. 2004 Nov 30;2(12):e422. doi: 10.1371/journal.pbio.0020422 (PMC532390; doi:10.1371/journal.pbio.0020422)
Supplement: Table S1 — Also available at http://genes.mit.edu/NielsenEtAl/. (4.3 MB ZIP). [file pbio.0020422.st001.zip › NielsenEtAl/html/916.html]

AN0688.1.NCU01328.1.MG02471.1.FG09998.1


```
 CLUSTAL W (1.82) Multiple Sequence Alignments - Introns Inserted


Sequence 1: NCU01328.1	692 aa
Sequence 2: FG09998.1	682 aa
Sequence 3: MG02471.1	687 aa
Sequence 4: AN0688.1	692 aa
Alignment Length: 702 aa
Number Identitical Residues: 462 aa
Alignment Score (without introns) 21003


MG02471.1 	-MGYSEVDKKAINTIRVLA0ADATFNANSGHPGAPM2GMAPVAHVLWDKIMKFNPKNPSW
NCU01328.1	MSSFTETDQLAINTIRVLA0ADATAHANSGHPGAPM2GMAPVAHVLFNRFMNFNPKNPKW
FG09998.1 	-MGYGELDQKAINTIRVLA0ADATAHSNSGHPGAPM2GMAPVAHVLFNKFMNFNPKNPDW
AN0688.1  	-MGYTELDQLAINTIRLLA0VDATAKANSGHPGAPM~GMAPVAHVLFNKFMKFNPKNPEW
          	  .: * *: ******:** .*** ::********* *********::::*:******.*

MG02471.1 	LNRDRFVLS2--------~NGHGCMLQYALLHLFGYAVSMDDLKKFR0QVDSITPGHPEA
NCU01328.1	LNRDRFVLS2--------~NGHACMLQYALLHLYGYALTIDDLKAFR0RIDSITPGHPEA
FG09998.1 	LNRDRFVLS2--------~NGHGCMLQYAILHLFGYDLSIDDLKAFR0SVDSRTPGHPEA
AN0688.1  	ANRDRFVLS~YEAHFSCD2NGHGCMLQYALLHLFGYGISMDDLKAFR0QLDSITPGHPEA
          	 ********  .:  :.. ***.******:***:** :::**** **  :** *******

MG02471.1 	HDTPGIEVTTGPLGQGICNAVGLAMAQAHTAATFNKDGFNLIDNYTYCFLGDGCLMEGVS
NCU01328.1	HDTPGVEVTTGPLGQGISNAVGLAIAQAHTAAVFNKPGYDLVNNYTYCFLGDGCLMEGVS
FG09998.1 	HDTPGVEVTTGPLGQGISNAVGLAMAQAHTAATFNKDGFELVNNYTYSFLGDGCLMEGVS
AN0688.1  	HDTPGIEVTTGPLGQGFANAVGLAIAQAHSGGVFNKPGYNLFDNYTYCFFGDGCAMEGVA
          	*****:**********:.******:****:...*** *::*.:****.*:**** ****:

MG02471.1 	SEACSLAGHLQLGNLIAIWDDNHISID~GDTNQAFTEDVAKRYESYGWHVEVVKDGDNDL
NCU01328.1	SEACSLAGHLQLGNLIAIWDDNRITID~GDTNQAFTEDVLKRYESYGWHIVTVLDGDNNL
FG09998.1 	SEACSLAGHLQLGNLIAIWDDNQITID~GDTAVAFTEDVPKRYEAYGWHVIKVDDGDHDL
AN0688.1  	SEAASTAGHLKLGNLIAIYDDNHISID1GDTKCAFTEDVTKRFEAYGWHVVWVKDGDNDL
          	***.* ****:*******:***:*:** ***  ****** **:*:****:  * ***::*

MG02471.1 	EGIEAAIKKCQQVKDKPSLIQLRTIIGFGSTQQGTHGVHGSPLKADDLKQLKEKFGFNPD
NCU01328.1	DLIADAIKKAKEVTDKPTLIQLKTTIGFGSKKQGTHDVHGSPLKADDIKQLKEKFGFNPE
FG09998.1 	AAIEAAIKEAKSVTDKPTLIQLKTTIGFGSLNQGTHGVHGSPLKADDIKQLKEKWGFNPD
AN0688.1  	EGIEAAIKEAQAVKDKPSMIRLTTTIGFGSKLQGTGGVHGNPLKADDIEGVKKRFGFDPA
          	  *  ***:.: *.***::*:* * *****  *** .***.******:: :*:::**:* 

MG02471.1 	ESFAVPQEVYDLYNKKAAEGAALEQEWDQLFAKYGEQYKAEHDDLVRRQTGELPQGWEKN
NCU01328.1	ESFAVPQEVYDLCHKASAKGAAKEEEWNQLFAKYAEEFKAEHDDLIRRQKGELPEGWEKH
FG09998.1 	ESFAVPQEVYDFYGKRSSEGAAREQEWNQLLAKYKEQYPKEHADLVRRLTGELPEGWEKS
AN0688.1  	QSFVVPQQVYDLYHKHAEEGAAQEQEWNQLLQKYAGEYPNEHADLTRRLSGKLPEGWEKS
          	:**.***:***:  * : :*** *:**:**: **  ::  ** ** ** .*:**:**** 

MG02471.1 	LPTYTPSDPAIASRKLSETVLTKIEAAIPELVGGSADLTGSNLTRWKGAVDFQPPALKLG
NCU01328.1	LPVYTPADPAVASRKLSENVLNKIFEAVPELVGGSADLTGSNLTRWKGAIDFQPPATGLG
FG09998.1 	LPVYTPSDPAIASRKLSETVLAKIEGVIPELFGGSADLTGSNLTRWKEAVDFQPKSTGLG
AN0688.1  	LPVYKPSDPAIASRKLSEAVLEKIHSVIPELLSGSADLTGSNNTRWKNAVDFQPPEYGIG
          	**.*.*:***:******* ** **  .:***..********* **** *:****    :*

MG02471.1 	DSNYAGRYIRYGVREHGMGAIMNGLAAYGTILPFAGTFLNFVSYAAGAVRLSALSQVRMI
NCU01328.1	--TYAGRYFRFGVREHGMGAIMNGMAAYGTLIPYGGTFLNFVSYAAGAVRLSALSQVRAI
FG09998.1 	--DYSGRYVRYGVREHGMGAILNGLAAYGTILPYSGTFLNFVSYAAGSVRLSALSRVRTI
AN0688.1  	--EWSGRYLRYGVREHAMAAIMNGLAAYGTVIPAAGTFLNFVSYAAGAVRLSALSRVRAI
          	   ::***.*:*****.*.**:**:*****::* .************:*******:** *

MG02471.1 	WVATHDSIGLGEDGPTHQPIETLAHFRALPNCMVWRPADGNETSAAYYVALTSKHTPSII
NCU01328.1	WVATHDSIGLGEDGPTHQPIETLAHFRALPNCMVWRPADGNETSAAYYVALTSKHTPSIL
FG09998.1 	WVATHDSIGLGEDGPTHQPIETLAHFRALPNTMVWRPADGNETSGAYYVCLTSKHTPSII
AN0688.1  	HVATHDSIGLGEDGPTHQPIETLAHFRALPNCMVWRPADGNETSAAYYSALTAKHTPSIL
          	 ****************************** ************.*** .**:******:

MG02471.1 	ALSRQNLPQLAGSTIEKAIKGGYVAHEVEGAQVTLVSTGSEVGICIDAVKYLADNHNIKA
NCU01328.1	ALSRQNLPQLEGSTIEKAIKGGYVLHEVEGADVTLISTGSEVCIAIDAVKELKEKHNIKA
FG09998.1 	ALSRQNLPQLEGSTIEKAAKGGYVLHEVEGADITLVSTGSEVSIAVDAAKYLEEKKNIKA
AN0688.1  	ALTRQNLPQLENSSIEAALKGAYPVFEAADAKVTIISTGSEVSICIDAAKYLQEKHGVVA
          	**:******* .*:** * **.*  .*. .*.:*::****** *.:**.* * :::.: *

MG02471.1 	RVVSMPCFEVFDTQSKEYRLSVLPDGIPSMSVEVMSTMGWERYTHEQFGLNRFGASGPYK
NCU01328.1	RVVSMPCMEVFDAQPKDYRLSVLPDGIPSLSIEVMSTMGWEKYSHEQFGLNRFGASGAYL
FG09998.1 	RIVSMPCFEAFDLQDKEYRLSVLPDGVPSLSIEVMSTMGWERYTHEQFGINRFGASGAYK
AN0688.1  	RVVSIPCFEVFDAQDKEYRLKVLPDGIPILSVEVCSTMGWERYSHEQFGLNRFGASGPYK
          	*:**:**:*.** * *:***.*****:* :*:** ******:*:*****:*******.* 

MG02471.1 	D~VYK0------~KFEFTPEGIAKRAAATVDFWKDVPNVRSPINRAFQQII
NCU01328.1	D~VYK~ASIPLP0KFEFTPEGIAKRAIATIDFWKDVPNIRSPINRAFQQLI
FG09998.1 	D0---~------~KFEFTPEGIAKRAVATIDFWKDVPNVRSPINRAFQQII
AN0688.1  	E~VYA~------~KFEFTPEGISKRALATIDFYKGVP-VRSPINRAFQQIL
          	:            *********:*** **:**:*.** :**********::
```
